# A gaze-independent audiovisual brain-computer Interface for detecting awareness of patients with disorders of consciousness

**DOI:** 10.1186/s12883-018-1144-y

**Published:** 2018-10-09

**Authors:** Qiuyou Xie, Jiahui Pan, Yan Chen, Yanbin He, Xiaoxiao Ni, Jiechun Zhang, Fei Wang, Yuanqing Li, Ronghao Yu

**Affiliations:** 1 Coma Research Group, Centre for Hyperbaric Oxygen and Neurorehabilitation, Guangzhou General Hospital of Guangzhou Military Command, Guangzhou, 510010 China; 2 School of Software, South China Normal University, Guangzhou, 510641 China; 3 Center for Brain Computer Interfaces and Brain Information Processing, South China University of Technology, Guangzhou, 510640 China

**Keywords:** Audiovisual brain-computer interface (BCI), Event-related potential (ERP), Semantic congruency, Disorders of consciousness (DOC), Awareness detection

## Abstract

**Background:**

Currently, it is challenging to detect the awareness of patients who suffer disorders of consciousness (DOC). Brain-computer interfaces (BCIs), which do not depend on the behavioral response of patients, may serve for detecting the awareness in patients with DOC. However, we must develop effective BCIs for these patients because their ability to use BCIs does not as good as healthy users.

**Methods:**

Because patients with DOC generally do not exhibit eye movements, a gaze-independent audiovisual BCI is put forward in the study where semantically congruent and incongruent audiovisual number stimuli were sequentially presented to evoke event-related potentials (ERPs). Subjects were required to pay attention to congruent audiovisual stimuli (target) and ignore the incongruent audiovisual stimuli (non-target). The BCI system was evaluated by analyzing online and offline data from 10 healthy subjects followed by being applied to online awareness detection in 8 patients with DOC.

**Results:**

According to the results on healthy subjects, the audiovisual BCI system outperformed the corresponding auditory-only and visual-only systems. Multiple ERP components, including the P300, N400 and late positive complex (LPC), were observed using the audiovisual system, strengthening different brain responses to target stimuli and non-target stimuli. The results revealed the abilities of three of eight patients to follow commands and recognize numbers.

**Conclusions:**

This gaze-independent audiovisual BCI system represents a useful auxiliary bedside tool to detect the awareness of patients with DOC.

## Background

Brain-computer interfaces (BCIs) decode brain activities into computer control signals with the aim at providing a non-muscular communication pathway with external devices [1]. Among these brain activities, event-related potentials (ERPs) have been widely used in electroencephalography (EEG)-based BCI systems [2]. ERP BCIs use visual/auditory/tactile stimuli that correspond to control operations [3, 4]. The user selects an operation by focusing on the corresponding stimulus (target) while ignoring other stimuli (non-targets). For instance, the P300 speller described by Farwell and Donchin presented a selection of characters in a 6 × 6 matrix from a computer display [5]. The user was required to focus attention on the row and the column that contained the target character, while each row and column of the matrix flashed at random. In this case, the target character flashed with a probability of 0.167 (2/12). The visual P300 ERP elicited by the oddball was identified and translated into a character.

BCIs can potentially detect the awareness of patients with disorders of consciousness (DOC), such as unresponsive wakefulness syndrome (UWS, formerly known as the vegetative state [6]) and minimally conscious state (MCS). The UWS is defined by the preservation of spontaneous or stimulus-induced arousal without self or environmental awareness, whereas the MCS is characterized by the presence of inconsistent but discernible behaviors. Keystones in diagnosis lies in recovering the voluntary response, such as the ability to follow commands and functional use two different objects, which indicates emergence from the UWS and the MCS, respectively [7]. At present, the clinical diagnosis of patients with DOC is conducted on the basis of behavioral scales in general, such as the Coma Recovery Scale-Revised (CRS-R), which takes use of overt motor actions to external stimuli during observation [8]. However, in recent years, electroencephalography (EEG), functional magnetic resonance imaging (fMRI) and other neuroimaging methods have shown that misdiagnosis of patients with DOC who display a severe lack of motor function is possible [9]. For instance, Cruse et al. tested a motor imagery-related BCI with a group of 16 patients with UWS. Three of these patients achieved offline accuracies ranging from 61 to 78% during the motor imagery tasks [10]. Monti et al. instructed 54 patients (23 with UWS and 31 with MCS) to “imagine playing tennis” and “walk through houses” during an fMRI experiment and found that five (4 with UWS and 1 with MCS) were able to modulate their sensorimotor rhythms [11]. Recently, many BCI paradigms have been proposed for patients with DOC [12–16]. Lule et al. [13] proposed an auditory oddball EEG-based BCI paradigm based on data from 16 healthy subjects, 3 patients with UWS, 13 patients with MCS, and 2 patients with locked-in syndrome (LIS). One patient with MCS and one patient with LIS achieved significant offline accuracies over the chance level. In our previous study [17], we detected command following in eight patients with DOC (4 with UWS, 3 with MCS and 1 with LIS) using a visual hybrid P300 and SSVEP BCI, and successfully revealed that one UWS patient, one MCS patient and one LIS patient possessed residual awareness. However, the use of BCI for detecting awareness of patients with DOC remains in primary stage. These patients exhibit a generally weak BCI performance as they have a much lower cognitive ability than healthy individuals. Furthermore, substantial differences in EEG signals have been observed between patients with DOC and healthy individuals because of severe brain injuries in the patients. Therefore, many efforts should be taken for developing novel BCIs to enhance the performance of awareness detection.

For BCI-based awareness detection, an important issue lies in the type of stimulus modality. Up to now, most BCI studies have focused on unimodal (e.g., auditory-only or visual-only) stimuli. Compared to unimodal stimuli, congruent multisensory stimuli may activate additional neurons and result in faster behavioral responses and more accurate perception/recognition [18, 19]. However, multisensory stimulus paradigms have rarely received attentions in the field of BCIs [20]. In this study, we concentrated on the potential benefits of employing audiovisual stimuli to improve BCI performance. To the best of our knowledge, only three BCI studies focused on investigating audiovisual stimuli [21–23]. Belitski and colleagues compared different types of auditory-only, visual-only and audiovisual speller BCIs to assess their relative performance. Their experimental results involved 11 subjects reported that the positive effects of an audiovisual ERP-BCI paradigm compared with the corresponding visual-only and auditory-only variants [21]. Sellers and Donchin tested a P300-based BCI in the visual, auditory, and audiovisual modes, and reported that auditory mode exhibited a significantly worse classification accuracy compared with visual or audiovisual mode [22]. In our recent study [23], we designed an audiovisual BCI for detecting awareness of DOC patients, in which the audiovisual stimuli were semantically congruent visual and spoken numbers. The patients were required to give respond to target stimuli through following the instructions. According to the results regarding 8 healthy subjects, the use of the audiovisual BCI resulted in a better performance than the corresponding auditory-only or visual-only BCI, and multiple ERP components were strengthened by the audiovisual congruent target stimuli, which were useful for improving target detection. In the above audiovisual BCIs, two or more than two buttons were used in the GUIs. Thus, the GUIs were not completely gaze-independent.

**Fig. 1:**
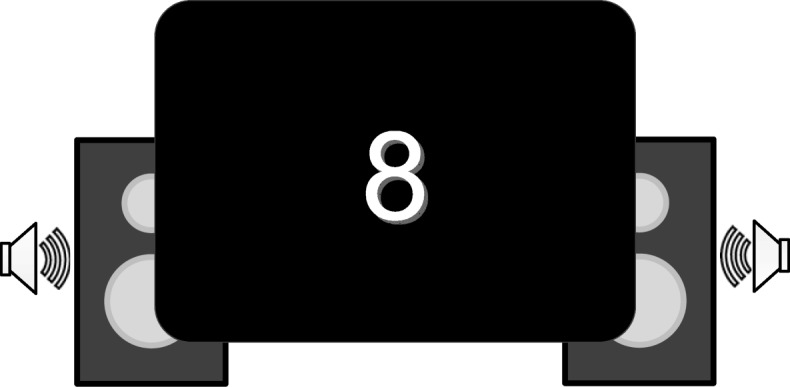
GUI of the audiovisual BCI. A pair of audiovisual stimuli were presented, which were semantically congruent (e.g., a visual number “8” and a spoken number “8”) or incongruent (e.g., a visual number “5” and a spoken number “6”)

Since most of the patients with DOC lack the ability to control eye movements, this study proposed a gaze-independent audiovisual BCI (Fig. 1) for detecting their awareness. Specifically, the stimuli included semantically congruent and incongruent audiovisual numbers. Furthermore, all audiovisual stimuli were presented individually, and thus the paradigm was completely gaze-independent. Ten healthy subjects and eight patients with DOC participated in our experiments. With this study, we aimed to (1) develop and validate a novel gaze-independent audiovisual BCI using semantically congruent and incongruent audiovisual stimuli; and (2) test whether this audiovisual BCI system assessed the covert conscious awareness of patients with DOC.

## Methods

### Subjects

The experiment included ten healthy subjects (nine males; average age ± SD: 29 ± 2 years) and eight patients with severe brain injuries (seven males; five with UWS and three with MCS; mean age ± SD: 42 ± 12 years; Table 1) in a local hospital. The recruitment was conducted based on pre-arranged inclusion/exclusion criteria. There were five inclusion criteria: 1) the patient had not taken centrally acting drugs; 2) the patient had not accepted sedation in the past 48 h; 3) the patient should keep eye opening for a period; 4) the patient had not suffered impaired visual or auditory acuity; 5) the patient had been diagnosed with VS or MCS after anoxic brain damage, traumatic brain injury (TBI), or cerebrovascular disease. There are three exclusion criteria: 1) the patient had a documented history of brain injury; 2) the patient once suffered an acute illness; 3) the patient had accepted hospitalization for less than 2 consecutive months. This study was approved by the Ethical Committee of the General Hospital of Guangzhou Military Command of PLA.

**Table 1:** Summary of patients’ clinical statuses. The clinical diagnosis listed in the brackets were obtained 1 month after the experiment

Patient	Age	Gender	Clinical Diagnosis	Etiology	Time Since Injury (months)	CRS-R Score (subscores)	
						Before the experiment	After 1 month
UWS1	34	M	UWS (UWS)	ABI	2	5 (1–1–1-1-0-1)	5 (1–1–1-1-0-1)
UWS2	55	M	UWS (MCS)	TBI	5	7 (1–1–2-2-0-1)	9 (1–1–4-2-0-1)
UWS3	41	M	UWS (UWS)	CVA	1	6 (1–1–1-1-0-2)	7 (1–1–2-1-0-2)
UWS4	48	M	UWS (MCS)	ABI	3	6 (1–1–2-1-0-1)	12 (1–3–5-1-0-1)
UWS5	22	M	UWS (UWS)	TBI	18	5 (1–1–1-1-0-1)	5 (1–1–1-1-0-1)
MCS1	53	F	MCS (MCS)	ABI	3	9 (1–3–2-1-0-2)	9 (1–3–2-1-0-2)
MCS2	37	M	MCS (EMCS)	TBI	4	8 (1–3–1-1-0-2)	19 (3–5–6-2-1-2)
MCS3	38	M	MCS (EMCS)	TBI	2	9 (1–3–2-1-0-2)	18 (3–5–6-1-1-2)

*ABI* anoxic brain injury, *CRS-R* Coma Recovery Scale-Revised, *CVA* cerebrovascular accident, and *TBI* traumatic brain injury, *CRS-R subscales* auditory, visual, motor, oromotor, communication, and arousal functions

UWS and MCS are diagnosed clinically on the basis of CRS-R. which contains 23 items organized in subscales that involve auditory, visual, motor, oromotor, communication, and arousal functions [24]. Each subscale is scored based on whether there is behavioral response to certain sensory stimuli defined in operation. For example, once the visual fixation of mirror occurs for more than 2 seconds at least twice in four directions, the score of visual subscale is 2 points, which means the patient exhibits MCS. Each patient participated in two CRS-R evaluations, one in the week before the experiment and another at 1 month after the experiment. Each evaluation contains five CRS-R assessments conducted in different days. The CRS-R scores for each patient presented in Table 1 were based on his/her best responses to the repeated CRS-R assessments.

### GUI and audiovisual paradigm

Figure 1 shows GUI employed in the study. A visual button was positioned in the central of a LED monitor (22 in.) (the area ration of button to monitor is 0:1). Two loudspeakers were located in the back of monitor which is used to show auditory stimuli. The visual stimuli consisted of 10 visual numbers (0, 1, …, 9), whereas the auditory stimuli included 10 spoken numbers (0, 1, …, 9; 22 kHz, 16 bit). The root mean square of power of all sound files was equalized to adjust sound intensities. Each stimulus presentation (300 ms) included a pair of the visual and spoken numbers that were semantically congruent (such as a visual number 8 and a spoken number 8) or incongruent (such as a visual number 5 and a spoken number 6). Furthermore, a 700-ms interval was employed between two consecutive stimulus appearances. Notably, all audiovisual stimuli are presented individually, with the visual stimuli appearing in the same location of the screen (the foveal visual field). Thus, the paradigm was gaze-independent.

Here, we used semantically congruent and incongruent audiovisual stimuli for two reasons. First, we constructed an oddball paradigm for evoking P300. Second, semantically congruent and incongruent audiovisual stimuli may produce more ERP components, such as the N400 and the late positive complex (LPC, also described as P600) [25–27], which might be useful for improving BCI performance.

### Experimental procedures

In the experiment, subjects seated comfortably in wheelchair and were required to avoid blinking eyes or moving bodies. The healthy subjects attended Experiment I, and the patients with DOC participated in Experiment II.

#### Experiment I

The experiment comprises three sessions which were performed randomly. The three sessions correspond to visual (V), auditory (A) as well as audiovisual (AV) stimulus, respectively. In each session, a calibration run of 10 trials was first employed to train the support vector machine (SVM) model, followed by an evaluation run of 40 trials. Notably, a small training dataset was collected specific to each subject, because this BCI system was designed mainly for patients with DOC who are easily fatigued during the experiment.

**Fig. 2:**
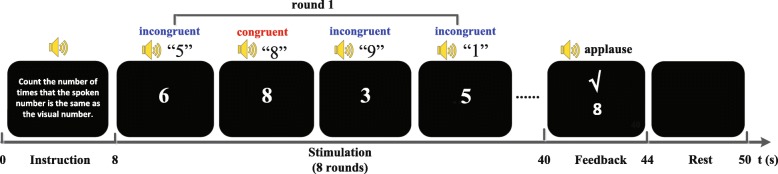
Procedure employed in one trial in the audiovisual condition, including audiovisual instruction (0–8 s), audiovisual stimulation (8–40 s), feedback on the classification result (40–44 s), and the rest period (6 s). The audiovisual stimulation involved eight presentations of four audiovisual stimuli (one semantically congruent and three semantically incongruent audiovisual number stimuli)

Figure 2 illustrates the experimenting process of one trail of audiovisual session. We firstly constructed four pairs of audiovisual stimuli where one pair was semantically congruent (such as a visual number 8 and a spoken number 8) and the other three pairs were semantically incongruent (such as a visual number 5 and a spoken number 6). Under the condition of semantic congruency/incongruency, these visual stimuli and auditory stimuli were pseudo-randomly chosen from the visual and spoken numbers (0, 1, …, 9). Each trial started as task instruction was presented visually and auditorily and lasted for 8 s. The instruction was “Count the number of times that the spoken number is the same as the visual number.” Following the presentation of the instruction, the four audiovisual stimulus pairs constructed as described above were individually presented 8 times in a random order. Specifically, four number buttons flashed from appearance to disappearance in a random order, with each appearance lasting 300 ms and with a 700-ms interval between two consecutive appearances. The appearance of a number button was accompanied by a spoken number for 300 ms. The subject was instructed to count the appearances of the congruent audiovisual stimuli (target) while ignoring the incongruent audiovisual stimuli (non-target). In this manner, the “oddball” effect was produced [28]. After 32 s, the BCI system performed online P300 detection for determining the audiovisual stimulus pair patients focused on. A feedback result determined by the BCI algorithm appeared in the center of the monitor. With correct result, positive audio feedback (applause) lasted for 4 s to encourage the subject. Otherwise, no feedback was presented and the screen was blank for 4 s. A short 6-s interval between two consecutive trials was utilized. Since four pairs of audiovisual stimuli were presented, one of which was the target, the chance level of accurate detection was 25%.

The experimental process of visual session and auditory session exhibited a similarity to audiovisual session, and there were two obvious exceptions. First, the instruction was “Focus on the target number (e.g., 8), and count the presenting times of target number”. Second, visual session used visual-only stimuli and auditory session adopted auditory-only stimuli.

#### Experiment II

Experiment II consisted of an audiovisual session in which each trial was conducted in the same procedure as the audiovisual session described in Experiment I. Eight patients with DOC participated in this experiment, in which 10 trials were calibrated and an online evaluation run of 40 trials were performed. Because patients were subject to fatigue, the calibration and evaluation runs were divided into five blocks, and each block contained 10 trials performed in different days. For these patients, the experiment lasted for 1–2 weeks. Based on the EEG data obtained in the calibration run, the SVM classifier on the first evaluation block was trained. For each of the later blocks, the classification model was updated using the data obtained from the previous block [12, 29]. For instance, data from Block 3 was used to update the SVM model for online classification of Block 4. During the experiment, the experimenters and families kept explaining these instructions to ensure that the patient concentrate themselves on audiovisual target stimuli. An experienced doctor carefully observed the patient to make sure the task engagement. Once the arousal degree was decreased (i.e., the patient closed eyes) or the patient kept moving body (e.g., severe eye blinking/moving) for over 4 s, the trail would be excluded. Additionally, according to the fatigue level of patients, the interval between two continuous trails was extended to at least 10 s.

### Data acquisition

A NuAmps device (Neuroscan, Compumedics Ltd., Victoria, Australia) was used to collect scalp EEG signals. Each patient was required to wear an EEG cap (LT 37) equipped with Ag-AgC1 electrodes. The EEG signals were referenced to the right mastoid. The EEG signals used for analysis were obtained from 32 electrodes which were positioned standardly in the 10–20 international system [30]. EEG signals over multiple trails were averaged, followed by the time lock, so as to identify ERPs. “HEOR” and “HEOL”, and “VEOU” and “VEOL” were used to record an electrooculogram (EOG). Then, a time-domain regression method which can record EOG was applied to reduce ocular artifacts. The impedances of all electrodes were maintained at less than 5μk. The EEG signals were amplified, sampled at 250 Hz and band-pass filtered between 0.1 Hz and 30 Hz.

### Data processing

#### Online detection algorithm

The same online analysis was performed for each session in Experiments I as well as II. We illustrated the online detection in an audiovisual session as an example. In term of each trial in the calibration and evaluation runs, EEG signals were first filtered at 0.1–20 Hz. The EEG signal epoch (0–900 ms after the stimulus onset) was extracted for each channel and stimulus appearance. This EEG epoch was down-sampled at a rate of 5 for obtaining a data vector which is composed of 45 data points to reduce computation of online processing. The vectors from all 30 channels were concatenated to obtain a new data vector of 1350 data points (45 × 30), which corresponded to a stimulus appearance. Second, we constructed a feature vector containing 1350 data points for each audiovisual stimulus pair by averaging the data vectors across the 8 appearances in a trial to improve the signal-to-noise ratio (SNR). Notably, these features contained several ERP components within 900 ms after stimulus onset. Third, we trained an SVM classifier by virtue of these feature vectors from calibration data. The SVM classifier was based on the popular LibSVM toolbox with the linear kernel. The parameters for the SVM were identified by five-fold cross-validation. Finally, for each online trial, we applied the SVM classifier for the four feature vectors (4×1350 data points) which corresponded to four pairs of audiovisual stimuli, thereby obtaining four SVM scores. The detection result obtained from this trial was determined as the audiovisual stimulus pair corresponding to the maximum of the SVM scores.

#### Offline data analysis for experiment I

We used the data from the evaluation run in each session of Experiment I to analyze the ERP. Specifically, after the band-pass filtering at 0.1–20 Hz, the EEG epochs of each channel were extracted from 100 ms pre-stimulus to 900 ms post-stimulus, and corrected baseline relying on the data from the 100-ms interval before stimulus. For artifact rejection, once the potential was larger than 60 μV in any channel, the epochs were excluded from averaging. The missing data for the ERP amplitudes were replaced with the mean value for the subject, as recommended for psychophysiological research [31]. During the evaluation run of each stimulus condition, we conducted time-lock averaging on EEG signals of 40 trials to extract the ERP responses.

The ERPs for target stimuli and non-target stimuli were compared to illustrate the effectiveness of the proposed audiovisual BCI paradigm. Specifically, a statistical analysis of the ERP components were conducted as described below [32–34]. First, based on the averaged ERP waveforms extracted above, the ERP components and their corresponding time windows were selected for all conditions. The width of the time window for each ERP component was 200 ms, as described in previous studies [26]. Then, the peak latency of each component was computed separately for each subject/condition. The latencies of maximum peaks were individually computed to ensure that the peak of each individual component was enclosed in its corresponding time window. Next, the mean amplitudes of these components were computed using a small window (50 ms in this study) surrounding the peak maximum. Finally, differences in the amplitudes of the signals between targets and non-targets were tested with two-way repeated measures analyses of variance (ANOVA) using the stimulus condition (the A, V, and AV conditions) and electrode site (“Pz”, “Cz”, and “Fz”) as within-subject factors for each of the ERP components. The missing data for ERP amplitudes were replaced with the mean value of the subject, as recommended for psychophysiological research [31]. Post hoc t-tests (Tukey’s test to correct for multiple comparisons) were further performed, when necessary. Results were considered significant when *p* values were less than 0.05.

#### Performance measures for experiment II

For each session, the accuracy represented the ratio of the number of all correct responses (hits) among the total number of presented trials. This study used two classes (hit and miss). A hit referred to the situation that output class of a trial was congruent stimulus (i.e. a true positive); otherwise, it was regarded as a miss (i.e. a false positive). Our paradigm employed four choices, namely, one congruent stimulus and three incongruent stimuli. The congruent stimulus (hit) exhibited a chance level of 25%, whereas the incongruent stimuli (miss) exhibited a chance level of 75%. A Jeffreys’ Beta distribution-based binomial test was used to compute the significance level of the four-class paradigm, which was expressed as follows [35]:

1 $$ \lambda =\left\{a+\frac{2\left(N-2m\right)z\sqrt{0.5}}{2N\left(N+3\right)}\right\}+z\sqrt{\frac{a\left(1-a\right)}{N+2.5}} $$

In the equation, N represents the number of trial, m represents the expected number of successful trial, *a* represents the expected accuracy (here it is 0.25), λ denotes the accuracy rate, and z denotes the z-score according to the standardized normal distribution. As one-sided test exhibited a significance level of 0.05, z is set as 1.65. Based on Eq. (1), the accuracy rate λ corresponding to the significance level (37.3% for 40 trials) was obtained.

## Results

### Results for healthy subjects

Ten healthy subjects participated in Experiment I. Table 2 summarizes the online classification accuracies for all healthy subjects. Among the visual-only, auditory-only, and audiovisual conditions, the online accuracy of auditory-only condition was the lowest for each healthy subject. For nine of the ten healthy subjects, the online audiovisual accuracy was larger than or equivalent to the visual-only online accuracy. The average online accuracies for all subjects were 92% for audiovisual condition, 84.75% for visual-only condition, and 74.75% for the audiovisual conditions (Table 2). A one-way repeated measures ANOVA was conducted to test the effect of the stimulus condition on the online accuracy. The stimulus condition exerted a significant effect (F_(2, 27)_ = 7.849, *p* = 0.005). Furthermore, according to the post hoc Tukey-corrected t-tests, online average accuracy for audiovisual condition was significantly higher compared with visual-only (*p* = 0.031 corrected) or auditory-only condition (*p* = 0.002 corrected).

**Table 2:** Online classification accuracies of the auditory-only (A), visual-only (V) and audiovisual (AV) sessions for healthy subjects

Subject	Accuracy (%)		
	A	V	AV
H1	75	80	90
H2	70	85	85
H3	55	85	85
H4	87.5	87.5	92.5
H5	70	80	90
H6	82.5	90	100
H7	67.5	80	100
H8	80	90	85
H9	82.5	87.5	97.5
H10	77.5	82.5	95
Average	74.75 ± 0.09	84.75 ± 0.04	92 ± 0.06

**Fig. 3:**
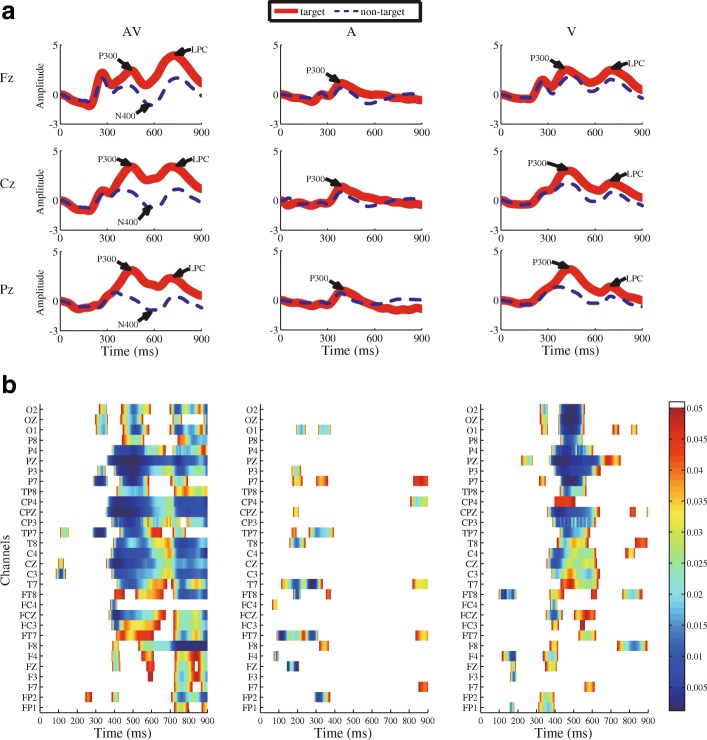
ERP waveforms and comparison of the results obtained from the audiovisual (AV, left panel), auditory-only (A, middle panel) and visual-only (V, right panel) conditions. **a** Average ERP waveforms of all healthy subjects recorded from the “Fz”, “Cz”, “Pz” electrodes. The solid and dashed curves correspond to the target and nontarget stimuli, respectively. **b** Point-wise running t-tests compared target with nontarget responses among all healthy subjects for 30 electrodes. Significant differences were plotted when data points met an alpha criterion of 0.05 with a cluster size greater than seven

We compared the brain responses evoked by target stimuli and non-target stimuli in the three conditions in our ERP analysis. The group mean ERP waveforms from 0 to 900 ms post-stimulus at the “Fz”, “Cz”, and “Pz” electrodes are shown in Fig. 3(a). Three ERP components, P300, N400, and LPC, were observed. We further determined the time windows for these ERP components (P300 window: 300–500 ms; N400 window: 500–700 ms; and LPC window: 700–900 ms). A two-way ANOVA did not reveal a significant interaction between the stimulus condition and electrode site for each of the ERP components. The electrode site did not exert a significant effect on each of the ERP components. However, according to the analysis, the stimulus condition can greatly affect each of the ERP components (P300: F_(2,63)_ = 7.928, *p* = 0.005; N400: F_(2,63)_ = 8.708, *p* = 0.004; LPC: F_(2,63)_ = 12.557, *p* = 0.003). Furthermore, post hoc Tukey-corrected t-tests revealed the following: (i) for the P300 component, the target and non-target stimuli in audiovisual condition delivered greater differences in amplitude from that in auditory-only condition (*p* = 0.003, corrected). (ii) For the N400 component, the greater differences in amplitude were observed between the target and non-target stimuli in audiovisual condition compared with visual-only (*p* = 0.031, corrected) or auditory-only condition (*p* = 0.006, corrected). (iii) For the LPC component, greater differences in amplitude were observed between the target and non-target stimuli in the audiovisual condition than in the visual-only (*p* = 0.004, corrected) or auditory-only condition (*p* = 0.002, corrected).

Point-wise running two-tailed t-tests were performed to evaluate the discriminative characteristics of target response and non-target response in the three conditions. From Fig. 3(b), certain time windows, such as 300–500 ms, 500–700 ms, and 700–900 ms, could show more discriminative characteristics in audiovisual condition compared with the other two conditions.

### Patients’ results

Eight patients attended Experiment II, with online results presented in Table 3. Three patients (UWS4, MCS2, and MCS3) exhibited an obviously higher accuracy (40–45%) than the chance level 25% (accuracy ≥37.3% or *p* < 0.05, binomial test). For patients UWS1, UWS2, UWS3, UWS5, and MCS1, the accuracies were not significant (i.e., ≤37.3%; ranging from 22.5 to 35%).

**Table 3:** Online accuracy of each patient

Subject	Trials	Hits	Accuracy	*p*-value
UWS1	40	11	27.5%	*p* = 0.7150
UWS2	40	9	22.5%	*p* = 0.7150
UWS3	40	12	30%	*p* = 0.4652
UWS4	40	16	**42.5%**	*p* = 0.0106
UWS5	40	13	32.5%	*p* = 0.2733
MCS1	40	14	35%	*p* = 0.1441
MCS2	40	16	**40%**	*p* = 0.0285
MCS3	40	18	**45%**	*p* = 0.0035

The accuracies that were significantly greater than the chance level 25% (accuracy ≥37.3% or *p* < 0.05) are highlighted in bold

**Fig. 4:**
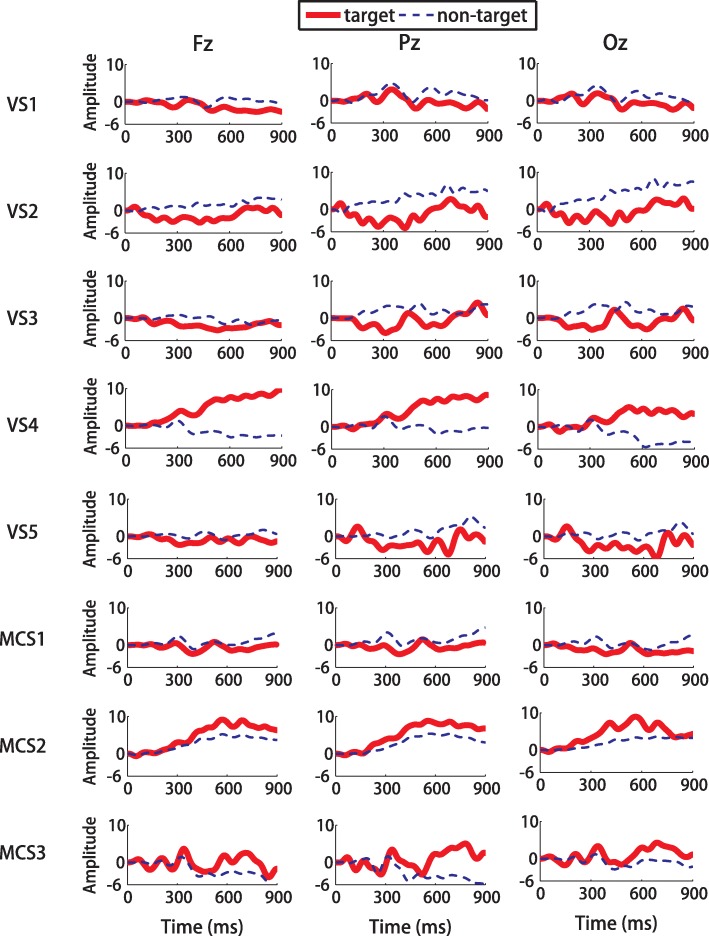
ERPs waveforms recorded from the “Fz”, “Cz” and “Pz” electrodes for the eight patients with DOC. The solid red curves correspond to the target stimuli, and the dashed blue curves correspond to the nontarget stimuli

The ERP waveforms were calculated for the eight patients with DOC. Specifically, the ERP waveforms in 0–900 ms post-stimulus were obtained by averaging the EEG channel signals across all 40 trials. Note that three trial epochs from patient UWS2 and two trial epochs from patients UWS5 and MCS2 were excluded from further data processing due to noise artifacts (the amplitude exceeded 60 μV). Fig. 4 presents the mean EEG signal amplitudes of eight patients recorded at “Fz”, “Cz” and “Pz” electrodes, with solid red curves representing target stimuli and dashed blue curves representing non-target stimuli. Furthermore, the meaningful ERP components were then determined for each patient. Since the ERP latencies were delayed in patients with acquired brain damage [36, 37], a wider time window of 300 ms (P300 window: 300–600 ms; N400 window: 500–800 ms; and LPC window: 700–1000 ms) was used for compensating the delayed latency of each ERP component in patients with DOC. If obvious positive/negative deflection emerged in the three time windows, the corresponding ERP component was elicited in this patient. For the three patients (UWS4, MCS2, and MCS3) who exhibited an obviously higher accuracy than the chance level, a P300-like component was apparent in each target curve, whereas the N400 and LPC responses were not evoked to the same extent as in the healthy controls. For the other five patients (UWS1, UWS2, UWS3, UWS5, and MCS1), none of the P300, N400, and LPC components were observed.

In the five entirely vegetative patients diagnosed by CRS-R assessments, patients UWS2 and UWS4 progressed to MCS 1 month after the experiment. Besides, patient UWS4 subsequently emerged from MCS in the follow-up (4 months after the experiment). Patients MCS2 and MCS3 subsequently emerged from their conditions and exhibited motor-dependent behavior 1 month after experiment. Other patients (UWS1, UWS3, UWS5, and MCS1) remained clinically unchanged at follow-up. More interestingly, according to the CRS-R, three patients (patients UWS4, MCS2 and MCS3) who obtained accuracy rates that were significantly higher than the chance level 25% (accuracy ≥37.3% or *p* < 0.05, binomial test), which greatly enhanced improved their consciousness levels to a large degree. Specifically, their CRS-R score of these patients improved from 6, 8, and 9 (before experiment) to 12, 19, and 18 (1 month after experiment), respectively.

## Discussion

In the present study, we developed a novel audiovisual BCI system using semantically congruent and incongruent audiovisual numerical stimuli. All audiovisual stimuli were presented sequentially, and thus the BCI system was gaze-independent. The experimental results obtained from ten healthy subjects indicted that the audiovisual BCI system achieved higher classification accuracy than the corresponding auditory-only and visual-only BCI systems. Furthermore, the audiovisual BCI was applied to detecting the awareness of DOC patients. Among the eight DOC patients (5 with UWS and 3 with MCS) who participated in the experiment, three (1 with UWS and 2 with MCS) achieved an obviously higher accuracy compared with the chance level (Table 3). According to our results, these three patients exhibited the abilities to follow commands and residual number recognition.

Here, our paradigm was unlike the standard oddball paradigms. The stimuli in our paradigm included semantically congruent and incongruent audiovisual numbers (25% congruent and 75% incongruent audiovisual stimuli), which were presented individually. This paradigm was applied in our experiment for healthy subjects, which indicated two main ERP correlates between the semantic processing (N400 and LPC) and the P300 component in the audiovisual condition. As shown in Fig. 3(a), the ERP responses to semantic processing first included a negative shift (N400) exhibiting a latency of 500–700 ms at electrodes “Fz”, “Cz” and “Pz” for semantically incongruent stimuli (nontarget). Then, a subsequent positive peak (LPC) was observed from 700 to 900 ms for semantically congruent stimuli (target) at electrodes “Fz”, “Cz” and “Pz”. These experimental results well fit previous reports focusing on semantic processing [38–40]. The time windows of P300, N400 and LPC are generally 200–400 ms, 400–600 ms, and 600–800 ms, respectively [26]. In the present study, the delayed latencies of ERP components might be due to the increased difficulty of the experimental task (i.e., distinguishing the semantically congruent audiovisual stimuli from the semantically incongruent stimuli). This finding was consistent with previous studies showing that an increase in the difficulty of the task results in prolonged latencies of ERP components [41, 42]. In our ERP analysis of healthy subjects, a stronger P300 response appeared in AV condition compared with A condition, and stronger responses for both N400 and LPC were detected in AV condition compared with A and V conditions. Furthermore, as shown in Fig. 3(b), in several time windows corresponding to the P300, N400 and LPC components, there was a greater difference between the target response and non-target responses for audiovisual condition compared with visual-only condition and auditory-only condition, which was helpful to improve the BCI performance (Table 2). Taken together, the potential benefits of our paradigm are described below. First, all the audiovisual stimuli were presented randomly in a serial manner to generate oddball effect. This gaze-independent oddball paradigm was partially supported by results from previous studies [43, 44]. Second, several ERP components, like the N400 and the LPC (Fig. 3), were enhanced using semantically congruent and incongruent audiovisual stimuli. The N400 component is a specific ERP component elicited by violations of a meaningful context [45]. Several studies used sentences or word-pair paradigms to record N400 components of semantic processing in patients with DOC [46, 47]. The LPC component was evoked in the semantic task, like memorizing congruous or incongruous auditory sentences [40, 48], memorizing words list [49, 50], as well as making decisions on congruency [51, 52]. In our audiovisual BCI system, we used all these enhanced ERP components and P300 for classification, which achieved performance improvement on proposed BCI in comparison by corresponding visual-only BCI and auditory-only BCI.

As reported earlier, behavioral observation scales such as CRS-R can yield a relatively high misdiagnosis rate in patients with DOC. BCIs represent an auxiliary bedside tool for detecting residual awareness of patients. Specifically, if the ability to follow commands and the experimental task-related cognitive functions appear in a UWS patient in virtue of a BCI system, we may conclude that the patient possesses awareness and a misdiagnosis might occur. In the present study, one UWS patient (UWS4) could implement the BCI experimental task accurately, which well fit previous fMRI [11] and EEG [53] data showing that some patients who are diagnosed with UWS based on the behavioral scales possessed residual cognitive functions and even exhibited consciousness to some extents. In fact, according to the behavioral CRS-R assessments, this patient with UWS progressed to MCS 1 month after the experiment and further emerged from MCS 3 months later. This behavioral observation supports the results of the conducted BCI assessment for the UWS patient.

Importantly, many cognitive functions, including the ability to understand instructions, selectively focusing on the target stimuli, and maintaining attentional focus on the target, are needed to perform the experimental tasks. One any abovementioned cognitive functions was missed, the experimental tasks may not be performed. Therefore, positive results in BCI experiments may indicate the existence of all these cognitive functions as well as residual awareness in these patients. However, negative results in BCI experiments should not be provided as final evidence for an absence of awareness, because even approximately 13% of healthy subjects exhibited BCI illiteracy, thus fail in effectively controlling a simple BCI [28].

In the study, DOC patients and healthy subjects exhibited a significant difference in ERP components (P300, N400 and LPC). For instance, the N400 or LPC responses were not evoked to the same extent in patients as in the healthy subjects (Fig. 4(a)). The main reason might be that the impairments of the brain networks might deteriorate the ability to evoke ERP components, such as N400 and LPC. Some patients might not simultaneously focus on the audiovisual stimuli, as observed for healthy subjects and patients UWS4, MCS2, and MCS3, and thus a neglect of the auditory or visual stimuli might also have allowed the deterioration of the evoked ERP components. Another reason lies in the consciousness fluctuation in DOC patients with time. ERP correlates in relation to semantic process cannot be effectively evoked due to low consciousness level. Besides, it is impossible to collect enough training data before each block as patients were fatigued, which may impact classifier performance. Actually, data of previous blocks collected in different days were used for updating the classifier of current block. The separation of calibration and evaluation sessions over several days might be affected by unreliable brain responses on different days. For instance, in a previous study [37], the authors proposed an auditory oddball paradigm for differentiating UWS patients and MCS patients. The study was performed at two different time points (labelled as T1 and T2), with the interval of at least 1 week. The presence of the P300 component at T1 did not well prove that it presented at T2. Based on these findings, DOC patients suffered a much lower BCI performance compared with healthy subjects, although many patients exhibited an obviously higher accuracy (about 45%) compared with the chance level.

It is necessary to conduct further studies for confirming the way to enhance the BCI performance for DOC patients. One potential solution is to update the SVM classifier using the online data with labels. During online awareness detection experiment, DOC patients with DOC were required to pay attention to a target, according to the instructions. Therefore, the labels for online data were available. Furthermore, the online data must be selected to ensure that patients were engaged in the task.

Our study, however, has several limitations. The first CRS-R evaluation was carried out before the experiment, and the experiment lasted from one to 2 weeks. Some patients were more responsive before than during the experiment. Moreover, our study lacks sensitivity because it requires a relatively high level of cognitive ability to understand task instructions.

## Conclusions

In summary, a gaze-independent audiovisual BCI was developed for detecting the awareness of patients with DOC in this study. Multiple ERP components, including the P300, N400 and LPC, were enhanced by the semantically congruent and incongruent audiovisual stimuli, which might be useful for improving audiovisual BCI performance. The audiovisual BCI system was first validated in ten healthy subjects and then applied for online detection of awareness of eight DOC patients. Our experimental results demonstrated both the abilities to follow commands and recognize residual number in three of the eight patients with DOC. The BCI system seems to be applicable for DOC patients with display both auditory and visual function. This audiovisual BCI paradigm should be extended to enable an application as simple “YES/NO” communication (e.g., patients focusing on the congruent audiovisual stimuli to communicate yes, and focusing on the incongruent audiovisual stimuli to communicate no) in this patient group.

## Abbreviations

ANOVA: Analysis of variance
BCI: Brain-computer interface
CRS-R: Coma recovery scale-revised
DOC: Disorders of consciousness
EEG: Electroencephalography
ERP: Event-related potential
fMRI: Functional magnetic resonance imaging
GCS: Glasgow coma scale
LIS: Locked-in syndrome
LPC: Late positive complex
MCS: Minimally conscious state
SVM: Support vector machine
UWS: Unresponsive wakefulness syndrome
VS: Vegetative state
